# Dietary Structure and Nutritional Status of Chinese Beekeepers: Demographic Health Survey

**DOI:** 10.2196/28726

**Published:** 2021-05-03

**Authors:** Boshi Wang, Zhangkai Jason Cheng, Qian Xu, Tiangang Zhu, Lin Su, Mingshan Xue, Lin Pei, Li Zhu, Peng Liu

**Affiliations:** 1 Department of Clinical Nutrition Peking University People's Hospital Beijing China; 2 State Key Laboratory of Respiratory Disease, National Clinical Research Center for Respiratory Disease The First Affiliated Hospital of Guangzhou Medical University Guangzhou China; 3 Wuhan Bao Chun Royal Jelly Co, Ltd Wuhan China

**Keywords:** beekeeper, body composition, nutrition survey, MMSE

## Abstract

**Background:**

Beekeeping and honey gathering are traditional forms of agricultural farming in China. However, only few studies have focused on the nutritional status and health level of this special occupational group.

**Objective:**

By comparing the health status of apiculturists (beekeepers) and vegetable farmers in plain areas of Hubei Province, and analyzing the influence of dietary structure and intake on their nutritional level, this paper provides a scientific theoretical basis for the further development of health education and disease prevention for beekeepers.

**Methods:**

From February to April 2016, 191/236 beekeepers (80.9% of the total beekeepers) with large-scale breeding (300-500 colonies) and 182 vegetable farmers in the same area were sampled by the cluster sampling method. Their nutrient composition was analyzed using a human body composition analyzer, dietary structure information was collected using the dietary frequency query method, and cognitive function was investigated. In addition, blood samples of both groups were collected.

**Results:**

A total of 362 valid questionnaires (beekeepers/vegetable farmers: 185/177) were collected, with an effective response rate of 97.1% (362/373). Both beekeepers and vegetable farmers were overweight, and the beekeepers’ grip strength was much stronger than that of the vegetable farmers’ regardless of gender. The dietary structure of beekeepers is very unique: 29.7% (55/185) of beekeepers indicated consuming royal jelly regularly for more than 10 years. Their main foods are grain, cereals, and fresh vegetables; 68.1% (126/185) of the beekeepers never drank milk and other dairy products, and their overall nutrient intake is unbalanced. The average intake of cellulose in this group was also significantly higher than that in the epidemiological survey in the same sex and age group. The intake of vitamin A and selenium in the beekeepers group was significantly higher than that in the vegetable-farmers group (all *P*<.001). The blood indices of creatinine (*P*=.03) and blood copper (*P*<.001) in the beekeepers group were significantly higher than those in the vegetable-farmers group, and the total protein, albumin, calcium, sodium, potassium, phosphorus, folic acid, and vitamin B12 in the beekeepers group were significantly lower than those in the vegetable-farmers group (*P*<.03 for potassium and *P*<.001 for others). The total Mini-Mental State Examination (MMSE) score of the beekeepers group was 28.1, significantly higher (*P*=.006) than that of the vegetable-farmers group (23.3).

**Conclusions:**

The beekeepers in this area have their special dietary structure, body nutrient level, and disease characteristics. The cognitive level of the beekeepers who regularly consume royal jelly is significantly higher than that of their peers. The chronic diseases of this special occupational group are closely related to their lifestyle and nutritional status, so more attention and in-depth studies are needed to improve the quality of life of this population.

## Introduction

China is a traditional beekeeping country, and has a long history of beekeeping. According to the statistics of the World Food and Agriculture Organization, in 2011, China was ranked number 1 in the world in terms of the number of bee colonies and the number of apiculturist practitioners. The country is also ranked number 1 in the production and export of bee products [1]. Bee products can be divided into 3 categories according to their formation and source: (1) products from bees (eg, honey, propolis, bee pollen); (2) bee secretions (eg, royal jelly, beeswax, bee venom); and (3) bodies of various insect states grown and developed by bees, such as bee pupae and bee larva, which are used as food accompaniment, bubble liquor, or processed into dry powder (capsules) [2]. Honey is produced by bees in honeycomb from nectar acquired from flowering plants. It is produced by worker bees through the action of amylase in salivary glands, and its main components are carbohydrates, proteins, minerals, vitamins, and phenols [3]. Royal jelly is a honey bee secretion used in the nutrition of larvae and adult queens. It is secreted by the hypopharyngeal glands and mandible glands of worker bees. Its active ingredients are more complex, among which 10-hydroxy-2-decenoic acid (10-HDA) is a unique unsaturated fatty acid, also known as royal jelly acid, which plays an immunosuppressive role by inhibiting the proliferation of splenic T cells and reducing the production of interleukin (IL)-12 by splenic dendritic cells [4].

Royal jelly protein is one of the important biologically active components during the development of queen bees, which can in vitro promote the expression of superoxide dismutase-1 (SOD1), and has an antiaging effect on human embryonic lung fibroblast (HFL-I) [5]. In addition, 3,10-dihydroxy decanoic acid, a fatty acid isolated from royal jelly, promotes IL-12 and IL-18, and inhibits IL-10, affecting the maturation and function of human monocyte–derived dendritic cells, contributing to the imbalance of antitumor and antiviral immune responses [6]. Other active ingredients, such as polypeptides, vitamins, flavonoids, and phytochemicals, can not only affect the cell metabolism process, but also provide important raw materials for the brain to synthesize glial cells, and also play a certain role in tissue damage repair [7]. However, most of the aforesaid studies are either animal experiments or in vitro cell experiments, and there is no clear medical evidence on whether royal jelly has an antidisease role after ingestion and how it is absorbed and utilized by the human body.

Hubei Province is located in the south of Central China and its middle region expands up to the Yangtze River. It is mainly distributed in the western mountainous area and the central and eastern plains. The climate is subtropical monsoon, with abundant heat and rainfall, sufficient illumination, distinct 4 seasons, and a long frost-free period [8]. In the plains, rapeseed and other crops are planted in a large area, and there are seasonal plants that provide a plentiful source of nectar, which is collected by honeybees [9]. The region has a long history of beekeeping which is handed down from one generation to another. It is a typical beekeeping province. In 2019, the total number of beekeeping colonies in Hubei Province was nearly 768,000. It is also the main province for processing and exporting bee products, with the intensive processing enterprises of bee products in Wuhan as the center, forming a sound production, processing, and marketing management system [9]. Huangpi District belongs to 1 of the 6 remote urban districts of Wuhan City, Hubei Province. Sanliqiao Street is located in the southern lakeside area of the district, where the cultivated land is deep, the soil is fertile, and the drainage and irrigation conditions are favorable. A number of farmers are converting the land to grow vegetables, which has become the “vegetable basket” of Wuhan citizens [10].

The author’s team found in the preliminary survey that because of the easy accessibility of work, more than half of the population regularly consume royal jelly. To explore the effects that differences in lifestyle and dietary structures have on the health status, this study compares beekeepers in Huangpi district with other regional vegetable farmers. Variables such as dietary structure, body composition, hematology indices, cognitive factors, and nutrition levels were compared and analyzed. This study provides the theoretical basis for health education and disease prevention for beekeepers’ community.

## Methods

### Survey Participants

This study adopts the method of cluster sampling, from February to April 2016. The study site is Huangpi district in Wuhan. A total of 191/236 beekeepers (80.9% of the total beekeepers) with large-scale breeding (300-500 colonies) and 182 vegetable farmers in the same area were sampled by the cluster sampling method. All participants were administered different questionnaires to collect demographic and nutritional information and underwent a physical examination to understand their current health status. A total of 362 questionnaires were collected (beekeepers group: 185; vegetable-farmers group: 177). The effective response rate is 97.1% (beekeepers group: 185/191, 96.9%; vegetable farmers group: 177/182, 97.3%). The criteria for inclusion were large-scale beekeeping with at least five years of practice, and no serious illness or communication problems. Informed consent was received from all participants in this study, and the consent form was signed.

### Survey Contents and Methods

#### Demographic Information

Data on age, gender, education level, years of royal jelly consumption, disease history (including self-reported disease history of respondents and diagnosis records of community health service centers/township health centers or medical and health institutions), and activities were collected.

#### Body Composition Analysis

The analysis was performed using the Eco I-BCA10 body composition analyzer (Beijing Sihai Huachen Science and Technology Co., Ltd.), and included physical examination of the participants (measurement of height, weight, waist circumference, hip circumference, grip strength, fatless weight, body fat percentage, bone mass, and muscle mass). Because of gender differences in body composition and grip strength, an intergroup comparison was conducted on the physical examination results of the beekeepers group and the vegetable-farmers group after adjusting gender factors.

#### Blood Index Test

For this purpose, whole blood samples from both groups were collected and stored at 4℃, and samples were sent to the Laboratory of Peking University People’s Hospital for unified testing within 1 week. Indicators included biochemical whole items (blood lipid, liver and kidney function, blood glucose, electrolytes, etc.), trace elements (iron, magnesium, copper, zinc), ferritin, folic acid, vitamin B12, and homocysteine. Ferritin, folic acid, and vitamin B12 were detected by a Roche E601 immunoanalyzer; trace elements by a Beckman AU5800 biochemical analyzer, and other indices by a Hitachi LST008 biochemical analyzer. The reagents were all tested using the original detection reagents by matching with the corresponding model.

#### Dietary Survey

The respondents were interviewed face to face by using a simplified version of the Food Frequency Inquiry questionnaire. The content of the questionnaire included the frequency and amount of food consumed (Multimedia Appendix 1).

#### Cognitive State Investigation

The Mini-Mental State Examination (MMSE) was developed in 1975, and is a standardized tool to rapidly screen individuals for cognitive dysfunction [11]. It includes 11 questions involving orientation, attention, immediate and short-term recall, language, and the ability to follow simple verbal and written commands. The total score is 30 points, and it takes only about 5-10 minutes to administer.

### Data Collection and Statistical Methods

#### Data Types

Descriptive epidemiological analysis methods include calculation rate, mean, etc. A *P* value of less than .05 indicates statistically significant differences. Among them, quantitative data such as waist circumference, bone mass, and grip strength (part of the physical examination index) after gender discrimination met the normal distribution and homogeneity of variance. Two independent samples *t* test (unpaired) were used to compare the differences in continuous variables between the beekeepers group and the vegetable-farmers group, whereas the chi-square test was used to compare the dichotomous variables. Blood index results (biochemical whole items, serum vitamins, and minerals, etc.) and cognitive function survey results were also compared using 2 independent samples *t* test (unpaired) without gender discrimination, and cognitive function differences were compared using regular intake of royal jelly as a grouping variable. To estimate food consumption frequency and intake, the recommended amount based on the Chinese balanced diet pagoda was taken as the standard [12] and its level was compared with the corresponding reference value. The data were statistically analyzed using SPSS software (version 26.0; IBM), and EpiData software (version 3.2; Pascal) was used to establish a database for all the collected questionnaire responses and a parallel double entry was performed. MATLAB R2021 (MathWorks) was used to produce the volcano plot. The dimension reduction method principal component analysis was applied to reduce the dimensionality of the blood index data set, while retaining most of the information.

#### Calculation of Daily Food Intake

The average daily intake of various kinds of food for each respondent in the past year was calculated according to the intake frequency and the amount of each intake. The basic formula is given as follows:

Food intake (g/day) = {intake frequency [times]/week × intake amount (g)}/7 days.

#### Calculation of Daily Nutrient Intake

Based on the Chinese Food Composition List 2009 (2nd Edition) compiled by the Nutrition and Food Safety of Chinese Center for Disease Control and Prevention [13], the average value of nutrients contained in the same category of food was taken to compile a food composition table, based on which the daily nutrient intake of each survey participant could be calculated. The formula is as follows:

Daily nutrient intake (g/day) = (food intake [g/day] × nutrient content in the cluster food composition table)/100 g.

### Ethical Statement

This study passed the ethical review of the Medical Ethics Committee of Peking University People’s Hospital (Approval Number: 2016PHB111-01). The research participants have been compensated with all tests free of charge and there were also gift compensations for daily supplies.

## Results

### General Results

There were 185 eligible individuals in the beekeepers group and 177 eligible individuals in the vegetable-farmers group. The smoking and drinking rates in the beekeepers group were higher than those in the vegetable-farmers group. More than half of the beekeepers have been consuming royal jelly for a long time or intermittently (55/185 beekeepers [29.7%] consumed royal jelly at least once a day for more than 10 years).

The prevalence of hyperlipidemia, stroke, and cardiovascular disease in the vegetable-farmers group was higher than that in the beekeepers group, and the prevalence of cardiovascular disease in the vegetable-farmers group (22/177, 12.4%) was 2.1 times higher than that in the beekeepers group (11/185, 6.0%). By contrast, the prevalence of hypertension and chronic gastritis was higher in the beekeepers group than that in the beekeepers group. Table 1 compares general results and the disease prevalence between the 2 groups.

**Table 1:** General results and disease prevalence rate between the beekeepers group and the vegetable-farmers group, sampled from Wuhan, China, between February and April 2016.

Characteristic	Beekeepers group (n=185)	Vegetable-farmers group (n=177)	*P* value
Male, n (%)	124 (67.0)	85 (48.0)	<.001
Female, n (%)	61 (33.0)	92 (52.0)	<.001
Mean (SD) age (years)	63.7 (7.9)	61.1 (9.8)	.39
Mean (SD) years of education	6.5 (0.8)	6.2 (0.8)	.28
Smoking, n (%)	62 (33.5)	45 (25.4)	.09
Drinking, n (%)	36 (19.5)	17 (9.6)	.008
Royal jelly consumption, n (%)	101 (54.6)	5 (2.8)	<.001
Hypertension, n (%)	80 (43.2)	70 (39.5)	.48
Hyperlipidemia, n (%)	11 (5.9)	20 (11.3)	.07
Cardiovascular disease, n (%)	11 (5.9)	22 (12.4)	.03
Stroke, n (%)	15 (8.1)	21 (11.9)	.23
Chronic gastritis, n (%)	40 (21.6)	25 (14.1)	.06

### Physical Examination Results

The hand grip strength of the beekeepers was much stronger than that of the vegetable farmers in both genders. Other physical examination indicators, such as height, weight, waist circumference, hip circumference, body fat percentage, and bone mass, were not significantly different between the 2 groups (Table 2). The *P* values have been adjusted by false-discovery rate (FDR).

**Table 2:** Physical examination results of the beekeepers group and the vegetable-farmers group, sampled from Wuhan, China, between February and April 2016.

Characteristic		Male				Female		
		Beekeepers group (n=124)	Vegetable-farmers group (n=85)	*P* value^a^	Beekeepers group (n=61)		Vegetable-farmers group (n=92)	*P* value^a^
Height (cm), mean (SD)		166.1 (5.0)	165.4 (6.4)	.76	154.9 (5.0)		154.4 (5.8)	.84
Weight (kg), mean (SD)		67.9 (8.7)	65.2 (9.3)	.76	59.4 (8.2)		59.1 (9.4)	.84
BMI (kg/m^2^), mean (SD)		24.6 (2.7)	23.8 (2.7)	.76	24.7 (3.3)		24.7 (3.5)	.54
Hip circumference (cm), mean (SD)		95.6 (4.7)	94.4 (9.5)	.48	95.7 (6.0)		97.1 (7.0)	.64
Fat-free mass (kg), mean (SD)		52.5 (5.6)	50.8 (9.3)	.70	39.4 (5.4)		39.7 (5.9)	.78
Waist circumference (cm), mean (SD)		85.7 (7.7)	86.2 (9.3)	.86	84.3 (8.5)		88.3 (8.1)	.84
Body fat rate (%), mean (SD)		21.4 (6.0)	19.7 (7.0)	.48	32.4 (6.6)		32.3 (6.8)	.78
Bone mass (kg), mean (SD)		2.8 (0.3)	2.8 (0.8)	.86	2.1 (0.3)		2.2 (0.3)	.84
Muscle mass (kg), mean (SD)		49.8 (5.4)	49.2 (5.7)	.86	37.3 (5.2)		37.4 (4.5)	.84
**Grip strength (kg)**								
	Dominant hand, mean (SD)	24.2 (2.6)	10.6 (4.8)	<.001	20.9 (3.8)		10.2 (4.0)	<.001
	Nondominant hand, mean (SD)	23.6 (2.7)	10.3 (4.8)	<.001	19.1 (4.4)		9.5 (3.7)	<.001

^a^False-discovery-rate adjusted *P* values; *P* values were obtained from *t* test.

### Blood Index Results

**Figure 1:**
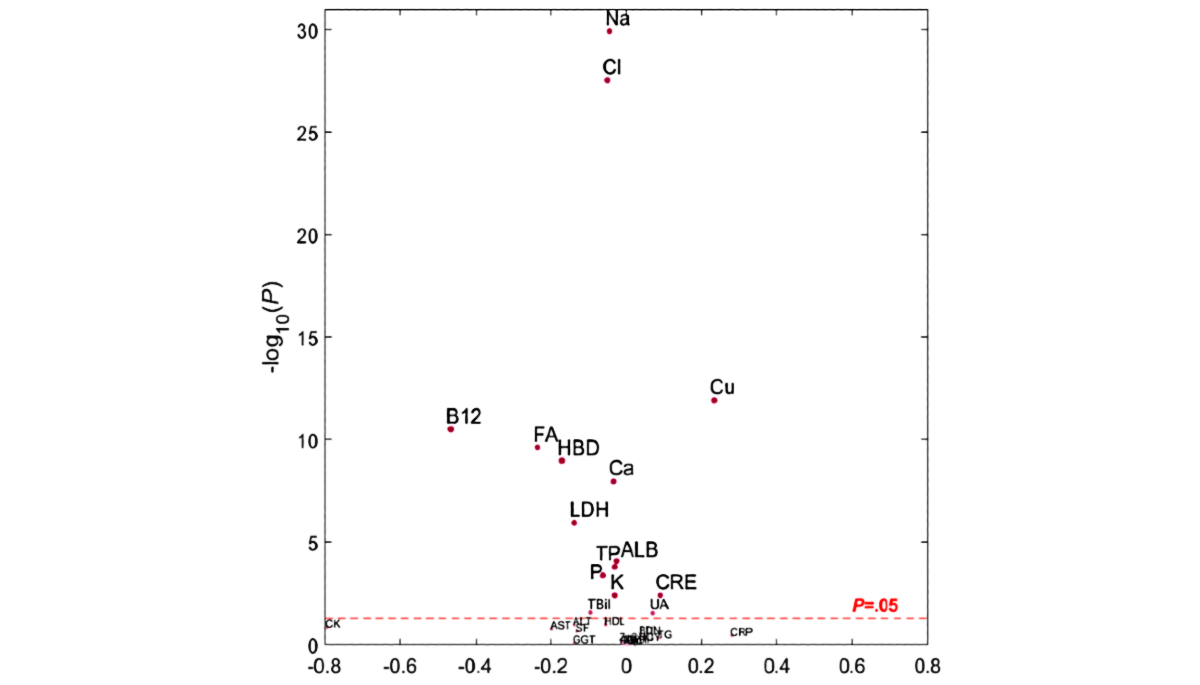
Volcano plot of blood index results, comparing between beekeepers group and vegetable-farmers group, sampled from Wuhan, China, between February and April 2016. Relative difference is positive if beekeepers group has higher value than vegetable-farmers group.

**Figure 2:**
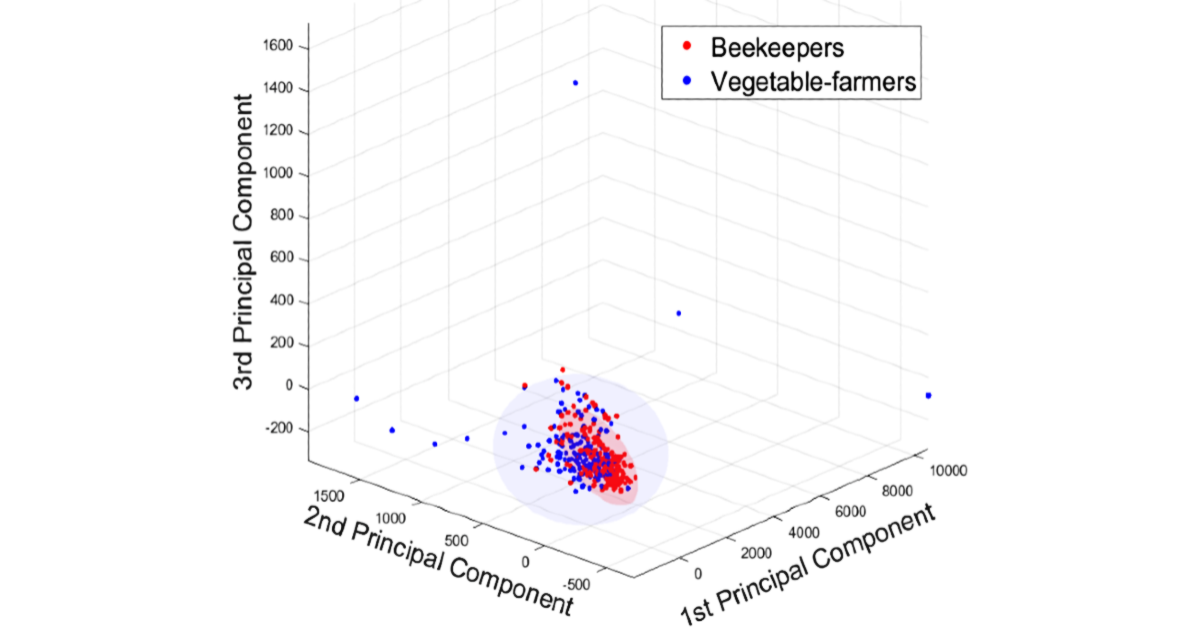
The first 3 principal components of blood index result, using PCA to compare between beekeepers group and vegetable-farmers group, sampled from Wuhan, China, between February and April 2016. Shaded areas represent two standard deviations of each population.

The main biochemical indices of the 2 groups were normal. Among them, the creatinine (*P*=.03) and uric acid (*P*=.02) levels of the beekeepers group were significantly higher than those of the vegetable-farmers group, and the total protein (*P*=.001) and albumin (*P*<.001) levels of the beekeepers group were significantly lower than those of the vegetable-farmers group. The results of serum vitamins and minerals test showed that all indices of the 2 groups were normal. Among them, the blood copper level in the beekeepers group was higher than that in the vegetable-farmers group, whereas calcium, sodium, potassium, phosphorus, folic acid, and vitamin B12 were lower than those in the vegetable-farmers group, and the differences were statistically significant (*P*<.03 for potassium and *P*<.001 for all others). Table 3 lists the main blood index results from both groups. Figure 1 is the volcano plot of the comparative results, showing the relative difference of each index between the 2 groups. We obtained the spread of points (beekeepers *vs* vegetable farmers) along the first 3 principal components in a principal component analysis plot (Figure 2). This result shows that beekeepers have a much closer spread of points compared with vegetable farmers, which indicates that the blood indices of beekeepers are similar, whereas those of vegetable farmers have bigger variation in value.

**Table 3:** Main blood indices of beekeepers and vegetable farmers, sampled from Wuhan, China, between February and April 2016.

Characteristic	Reference value, range	Beekeepers, mean (SEM)	Vegetable farmers, mean (SEM)	*P* value^a^
Glucose (mmol/L)	3.3-6.1	5.7 (0.1)	5.7 (0.1)	.90
Total cholesterol (mmol/L)	2.9-6.2	5.1 (0.7)	5 (0.7)	.90
Total triglyceride (mmol/L)	0.45-1.7	1.6 (0.1)	1.5 (0.1)	.90
Creatinine (mmol/L)	59-104	72.7 (1.4)	66.5 (1.3)	.03
Uric acid (μmol/L)	208-428	358.7 (6.5)	334.3 (7.2)	.02
High-density lipoprotein cholesterol (mmol/L)	1.03-1.55	1.46 (0.03)	1.54 (0.03)	.90
Low-density lipoprotein cholesterol (mmol/L)	1.9-4.1	2.82 (0.06)	2.71 (0.06)	.90
Homocysteine (μmol/L)	0.0-15	14.4 (0.4)	13.8 (0.6)	.90
Potassium (mmol/L)	3.5-5.3	4.1 (0.03)	4.3 (0.03)	.03
Sodium (mmol/L)	137-147	137.1 (0.4)	143.4 (0.1)	<.001
Calcium (mmol/L)	2.2-2.65	2.27 (0.01)	2.35 (0.01)	<.001
Phosphorous (mmol/L)	0.81-1.45	1.02 (0.1)	1.01 (0.01)	<.001
Iron (μmol/L)	7.9-34.4	20.0 (0.5)	20.1 (0.5)	.90
Magnesium (mmol/L)	0.7-1.05	0.93 (0.01)	0.93 (0.01)	.90
Copper (μmol/L)	11-24	14.8 (0.3)	11.7 (0.3)	.001
Zinc (μmol/L)	10.7-17.7	14.5 (0.2)	14.8 (0.2)	.90
Serum ferritin (ng/mL)	30-400	137 (8)	157 (9)	.90
Total protein (g/L)	65-85	71.8 (0.4)	74.1 (0.4)	.001
Albumin (g/L)	40-55	44.0 (0.2)	45.2 (0.2)	<.001
Folic acid (ng/mL)	4.2-19.8	9.7 (0.3)	12.3 (0.3)	<.001
Vitamin B12 (pg/mL)	197-771	290 (11)	467 (22)	<.001

^a^False-discovery rate–adjusted values.

### Frequency of Distribution of Various Food Intake

Among the surveyed beekeepers and vegetable farmers, the daily diet included grain cereals (185/185 [100%] and 177/177 [100%], respectively) and fresh vegetables (178/185 [96.2%] and 167/177 [94.4%], respectively). The frequency of intake of beef, mutton, and chicken was very low. About 63.2% (117/185) of beekeepers and 93.8% (166/177) of vegetable farmers never ate beef and mutton. At the same time, 68.1% (126/185) of beekeepers and 72.3% (128/177) of vegetable farmers never drank milk.

### Daily Intake Results of Various Foods

The intake of meat, fish, shrimp, eggs, milk, fruit, and bean products was lower than the recommended dietary pagoda for more than 50% of the individuals in both groups (92/185 beekeepers and 88/177 vegetable-farmers). About 70.3% (130/185) of the beekeepers and 82.5% (146/177) of the vegetable farmers consumed too much cooking oil (mean [SD]): 44.8 (25.0 g/day) in the beekeepers group and 55.2 (28.0 g/day) in the vegetable-farmers group.

### Daily Energy and Thermogenic Nutrient Intake Results

The daily energy and protein intake of beekeepers and vegetable farmers was low for both men and women (Table 4). The beekeepers had a reasonable proportion of the 3 heat-producing nutrients, whereas the vegetable farmers had a low calorific proportion of protein and carbohydrate (the percentage of calories provided by protein and carbohydrate to total calories), and a high calorific proportion of fat.

**Table 4:** Mean daily energy and intake of 3 major heat-producing nutrients of beekeepers and vegetable farmers from Wuhan, China (sampled period February-April 2016).

Sex and characteristic		Energy (kilocalorie)		Protein (g)		Fat (g)		Carbohydrate (g)	
		Beekeepers	Vegetable farmers	Beekeepers	Vegetable farmers	Beekeepers	Vegetable farmers	Beekeepers	Vegetable farmers
**Male**									
	Intake, mean (SD)	1567 (23)	1516 (39)	62 (1)	57 (2)	63 (1)	64 (3)	193 (4)	183 (5)
	Calorific proportion (%)	N/A^a^	N/A	15.9	14.9	36	37.9	49.3	48.4
**Female**									
	Intake, mean (SD)	1396 (25)	1754 (40)	53 (1)	56 (5)	60 (1)	85 (6)	166 (4)	196 (15)
	Calorific proportion (%)	N/A	N/A	15.2	12.8	29.9	33.9	47.7	44.8
Reference calorific proportion (%)		N/A	N/A	15-20	N/A	25-30	N/A	50-60	N/A

^a^N/A: Not applicable

### Intake of Other Nutrients

The intake of vitamin A (*P*<.001) and selenium (*P*<.001) in the beekeepers group was higher than that in the vegetable-farmers group, and the difference was statistically significant, whereas there was no significant difference in other nutrients between the 2 groups (Table 5).

**Table 5:** Comparing average daily intakes for other nutrients between beekeepers and vegetable farmers from Wuhan, China (sampled period February-April 2016).

Nutrient	Beekeeper male		Beekeeper female		Vegetable farmer male			Vegetable farmer female			*P* value	
	Intake (g/day), mean (SD)	Dietary reference intakes, %	Intake (g/day), mean (SD)	Dietary reference intakes, %	Intake (g/day), mean (SD)	Dietary reference intakes, %	Intake (g/day), mean (SD)		Dietary reference intakes, %			
Cellulose (g)	18.2 (5.7)	72.8	17.5 (5.2)	70	18.4 (7.4)	74	20.4 (21.9)		82			.96
Vitamin A (µg)	249.2 (181.4)	44.5	165.6 (136.4)	28.4	194.8 (201.2)	24.2	127.3 (208.5)		18.1			<.001
Vitamin B1 (mg)	0.8 (0.2)	57.1	0.7 (0.2)	58.3	0.7 (0.2)	54.2	0.7 (0.8)		65.8			.39
Vitamin B2 (mg)	1.1 (0.3)	78.6	1.0 (0.3)	83.3	1.1 (0.4)	81	1.1 (1.2)		98.3			.14
Vitamin C (mg)	190.5 (90.1)	190.5	197.1 (80.8)	197.1	209.7 (107.0)	210	238.6 (258.5)		238			.77
Total vitamin E (mg)	32.1 (9.1)	N/A^a^	31.7 (7.7)	N/A	32.3 (15.3)	N/A	43.9 (41.6)		N/A	.96		
Folic acid (µg)	634.2 (237.7)	158.6	623.4 (215.9)	155.9	666.9 (305.3)	167	737.5 (787.9)		184.2			.42
Potassium (mg)	2093.2 (649.4)	104.7	1988.4 (620.9)	99.4	2101.2 (889.2)	105	2265.6 (2432.6)		113.2			.53
Calcium (mg)	704.3 (255.2)	70.4	692.3 (237.2)	69.2	730.5 (339.6)	73	807.6 (860.5)		80.7			.68
Magnesium (mg)	348.9 (89.0)	105.7	319.3 (86.0)	96.7	341.8 (127.6)	103.3	368.1 (379.9)		111.5			.65
Iron (mg)	27.3 (7.4)	227.5	24.8 (6.7)	206.7	26.6 (10.0)	222	28.1 (29.4)		233.3			.66
Manganese (mg)	6.3 (1.6)	140	5.7 (1.5)	126.7	6.1 (2.2)	135.5	6.7 (6.7)		148.8			.88
Zinc (mg)	11.0 (2.5)	88	9.8 (2.5)	130.7	10.5 (3.7)	84	11.2 (11.5)		149.3			.39
Copper (mg)	1.9 (0.5)	237.5	1.7 (0.5)	212.5	1.7 (0.6)	221.2	1.6 (2.0)		237.5			.93
Phosphorus (mg)	904.1 (218.0)	125.6	791.6 (224.2)	109.9	860.1 (332.2)	119.4	887.0 (942.0)		123.1			.13
Selenium (µg)	41.8 (13.3)	69.7	35.0 (12.3)	58.3	38.8 (18.4)	64.6	38.3 (42.9)		63.3			<.001

^a^N/A: Not applicable.

### Results of Cognitive Function Survey

As presented in Table 6, the total MMSE score of the beekeepers group was 28.1, which was significantly higher (*P*<.001) than that of the vegetable-farmers group (23.3). Among them, the beekeepers group showed significantly better cognitive performance in Auditory Verbal Learning Test (*P*=.01), Clock Drawing Test (*P*=.005), and Verbal Fluency Test (*P*=.005). The 2 groups of people were further divided into 2 subgroups according to whether they regularly consumed royal jelly or not. It was concluded that the total MMSE score of the subgroup consuming royal jelly for several years was significantly higher than that of the subgroup not consuming royal jelly (*P*=.006).

**Table 6:** Results of cognitive function survey in beekeepers and vegetable farmers from Wuhan, China (sampled between February and April 2016).

Test	Beekeepers			Vegetable farmers			*P* value		Royal jelly group			No royal jelly group			*P* value
	N	Score, mean (SD)	N		Score, mean (SD)			N		Score, mean (SD)	N		Score, mean (SD)		
Total Mini-Mental State Examination	180	28.1 (3.5)	165		23.3 (5.5)	<.001		100		25.5 (3.4)	245		23.7 (5.0)	.006	
Rey Auditory Verbal Learning Test	185	30.7 (13.4)	177		26.1 (13.6)	.01		106		30.0 (14.5)	256		27.1 (13.4)	.07	
Delayed word recall test	185	5.3 (3.8)	177		4.1 (3.6)	.15		106		4.9 (3.8)	256		5.0 (3.6)	.66	
Clock drawing test	180	3.8 (1.5)	158		3.1 (1.9)	.005		99		3.9 (1.5)	239		3.3 (1.8)	<.001	
Verbal fluency test	182	14.1 (4.7)	160		12.0 (3.9)	.005		101		14.5 (5.2)	241		12.5 (4.0)	<.001	

## Discussion

### Principal Findings

This study is the first to carry out a comprehensive epidemiological investigation on beekeepers. This population has a unique dietary structure, living habits, and disease distribution characteristics; additionally, its overall cognitive level is significantly higher than that of vegetable farmers from the same area. It is worth noting that the average age of the beekeepers and vegetable farmers investigated in this study was over 60, indicating the aging population of beekeepers, which is consistent with the research results of the National Bee Industry Technology System [14].

It is difficult to attract young labor force into the beekeeping industry, possibly due to poor beekeeping conditions, the need to chase flowers in the wild for honey, beekeeping facilities that are relatively backward, a low degree of mechanization, and a high physical consumption. The results showed that the rates of smoking and drinking were higher among beekeepers than those among vegetable farmers, which may be related to the higher proportion of males in the beekeepers group, compared with that in the vegetable-farmers group. A large number of studies have shown that overweight and obesity are risk factors for hypertension and cardiovascular diseases in the elderly. The Chinese Hypertension Survey [15] showed that the prevalence of hypertension in people aged 55-64 was 44.3%, and other scholars have concluded through meta-analysis that the prevalence of hypertension in rural areas in southern China was 20.2% [16]. According to the BMI classification standard of adults in China, the BMI of the beekeepers group and that of the vegetable-farmers group was in the “overweight” category. Waist circumference and body fat rate also exceeded the standard of normal adults in China [17], but these did not reach the diagnostic standard of obesity. Thus, it is not difficult to understand the prevalence of hypertension in both groups, which was significantly higher than 20.2% (identified in 80/185 [43.2%] beekeepers and 70/177 [39.5%] vegetable farmers). As hypertension is an independent risk factor of stroke, the prevalence of stroke (identified in 15/185 [8.1%] beekeepers and 21/177 [11.9%] vegetable farmers) is also significantly higher than the national average (2.06%) [18]. At the same time, the physical examination results showed that both men and women in the beekeepers group had a much stronger hand grip than those in the vegetable-farmers group. This significant difference in grip strength is likely due to the difference in labor intensity between the 2 groups: beekeepers need to follow the blooming period all year round, with trucks used to carry beehives from one flower field to the next. This kind of work characteristic is also common among the beekeepers in other areas of China. As a result of convenient transportation and rapid development of agricultural mechanization modernization, the labor intensity of vegetable farmers in the flat terrains of Huangpi District is far lower than that of the beekeepers.

Nearly 29.7% (5/185) of beekeepers reported consuming royal jelly regularly for more than 10 years, which is significantly higher than the proportion consumed by the common population and vegetable farmers in the same area. This is no surprise, as it is easier to obtain royal jelly due to the easy accessibility of work. Compared with vegetable farmers, beekeepers had higher intake of fish food, which mainly included preserved and dried fish. This might be mainly related to the special occupational requirements, whether fixed or transfer operations, of beekeepers, who need to work continuously in the rare natural environment for 5-8 months and keep up with the change in the flowering period [9]. The hard-working conditions in different provinces and regions might have resulted in the difficulty to obtain meat, soy products, eggs, milk, and other foods, while pickled vegetables, dried fish, cooking oil, and other food are easier to carry and eat over longer distances. This directly led to the insufficient intake of protein, most vitamins, calcium, zinc, and other nutrients in the beekeepers group. Moreover, salt in such preserved foods is well over standard limits. Long-term consumption of salted foods remains a risk factor for hypertension and chronic gastritis [19]. Whether this leads to a higher incidence of these 2 diseases among beekeepers remains to be further studied. In addition, the dietary structure has certain regional characteristics. Hubei is a land of fish and rice. Pickled fish, meat, rice, and other foods are popular in the province; however, seafood and dairy products are not popular enough [10,14]. Therefore, 68.1% (126/185) of the beekeepers and 72.3% (128/177) of the vegetable farmers in the study never drank milk. Although the average intake of cellulose in both groups were lower than the recommended amount (25 g) in China [12], it was significantly higher than the results of the epidemiological survey in the same gender and age group, which may be related to the fact that the vegetable supply is sufficient, and the daily diet of the residents mainly includes fresh vegetables and cereals [8].

The results of this study showed that the main biochemical indices of the 2 groups are normal, but the uric acid level of the beekeepers group was significantly higher than that of the vegetable-farmers group, and was close to the high uric acid level (360 μmol/L), which was speculated to be related to the long-term administration of royal jelly. In general, fructose and glucose account for 90% of total sugar in royal jelly. High consumption of fructose-containing products can lead to the accumulation of phosphorylated products and continuous consumption causes liver ATP (adenosine triphosphate) to produce a large amount of uric acid through metabolism. At the same time, the serum levels of folic acid (vitamin B9) and vitamin B12 in the beekeepers group were lower than those in the vegetable-farmers group, possibly because the B vitamins in royal jellies were mainly B5 (52.8 mg/100 g) and niacin (42.4 mg/100g), whereas the contents of folic acid (0.4 mg/100 g) and vitamin B12 (0.2 mg/100 g) were very low [20]. Although the mineral content in honey varies depending on plant sources, the mineral content in royal jelly is relatively stable, accounting for about 1.5% [21]. Royal jelly is considered to be a homeostasis-regulated larval lactation form that is generally harvested commercially after 4 days of age for queen bee larvae [22]. The blood indices of beekeepers were more concentrated than those of vegetable farmers, and the number of people exceeding the normal value was less, indicating that beekeepers were healthier than vegetable farmers. Possible reasons for this are the small lifestyle differences as well as more similar and healthier dietary structure and eating habits among beekeepers. Whether it is related to the long-term consumption of royal jelly needs further study and verification. Al-Kahtani and Taha [23] found that, due to different harvesting times, royal jellies harvested 24 hours after the hatching of larvae had the highest contents of phosphorus and zinc; 48 hours later, the contents of calcium, potassium, and sodium were the highest; 96 hours later, the content of copper was higher. Combined with the findings of this study, we suggest that the levels of different mineral nutrients in the body can be improved by consuming royal jelly at different harvest times.

In addition, the intake of vitamin C and folic acid was significantly higher than that of dietary reference intakes, and the intake of vitamin A and selenium was significantly higher than that of vegetable farmers. The worker bees must use honey and pollen as raw materials to produce royal jelly. The nectar source and powder source plants (ie, plants that provide pollen, such as rape and locust flowers) vary based on regions and soil composition; consequently, the mineral types and contents in the royal jelly produced are also different [24]. There are many selenium-rich zones in Hubei Province [25], and oilseed rape, as a cruciferous plant, has a strong ability to enrich selenium [26]. Therefore, we speculated that the royal jelly eaten by beekeepers in this study may have a higher selenium content. Selenium plays a certain role in the nutritional value of royal jelly, but as a micronutrient, some scholars believe that compared with potassium (K), sodium (Na), calcium (Ca), magnesium (Mg), and other elements, the content of selenium in royal jelly is very low [27]. Therefore, with the development of instrumental analysis technology, it is necessary to further compare the selenium content of royal jelly produced in different regions in future studies.

When screening for cognitive function, the total MMSE score of the beekeepers group was significantly higher than that of the vegetable-farmers group, and the cognitive performance of the beekeepers who regularly consumed royal jelly on auditory word learning, clock drawing test, and language fluency was better than that of the beekeepers and vegetable farmers who did not consume royal jelly. Studies have shown that brain-derived neurotrophic factor (BDNF), especially BDNF in the hippocampus, can regulate learning and memory processes through interaction with TrkB receptors, such as long-term enhancement, synaptic plasticity, axonal budding, and dendritic proliferation [28]. As a small unsaturated fatty acid, 10-HDA in royal jellies can pass through the blood–brain barrier and have effects similar to BDNF [29]. Alzheimer disease (AD) has complex causes, including nerve fiber tangles, amyloid beta deposition, inflammatory response, and oxidative stress response. In many animal experiments at home and abroad and in vitro cell experiments, it has been proved that royal jelly can improve the spatial learning and memory ability of AD model rats [30] by effectively alleviating the toxicity of β-amyloid in AD and significantly reducing β-amyloid species [31]. However, whether royal jelly and its functional components have a positive effect on the prevention and delaying of human AD and their possible mechanism of action remain to be further studied.

### Study Limitation

A limitation of this study is that only the beekeepers in a certain area of Central China were taken as the research subject, which has certain regional limitations and needs to be further investigated and studied in more areas. Moreover, the participants’ recollections of how much they ate could be skewed.

### Conclusions

To sum up, combined with the results of this survey, beekeepers in Hubei Province have their special dietary structure, human body composition, and disease characteristics. The lifestyle and nutritional status of beekeepers, a special occupational group, are closely related to their chronic diseases, which need more attention and in-depth study. The role of royal jelly in the prevention and treatment of dementia and its related mechanisms will become the focus of our future research.

## Appendix

Multimedia Appendix 1 Food frequency, intake, and lifestyle questionnaire.

## Abbreviations

10-HAD: 10-hydroxy-2-decenoic acid
AD: Alzheimer disease
BDNF: brain-derived neurotrophic factor
FDR: false-discovery rate
IL: interleukin
MMSE: Mini-Mental State Examination
SOD-1: superoxide dismutase-1
