# Supplementary material for: Dietary Structure and Nutritional Status of Chinese Beekeepers: Demographic Health Survey
Source: JMIR Public Health Surveill. 2021 May 3;7(5):e28726. doi: 10.2196/28726 (PMC8129875; doi:10.2196/28726)
Supplement: Multimedia Appendix 1 [file publichealth_v7i5e28726_app1.docx]

| **Supplementary S1**  **Food frequency, intake and lifestyle questionnaire** | | | | |  | |  | | |
| --- | --- | --- | --- | --- | --- | --- | --- | --- | --- |
| Dietary survey | |  |  | | | | | |  |
| 1. Do you eat pork? | No； _____ time(s) per week | | | | | | | □ | E1 |
| Quantity per consumption | <50g; 50-100g; >100g | | | | | | | □ | E2 |
| 1. Do you eat lamb? | No； _____ time(s) per week | | | | | | | □ | E3 |
| Quantity per consumption | <50g; 50-100g; >100g | | | | | | | □ | E4 |
| 1. Do you eat chicken/duck? | No； _____ time(s) per week | | | | | | | □ | E5 |
| Quantity per consumption | <50g; 50-100g; >100g | | | | | | | □ | E6 |
| 1. Do you eat fish? | No； _____ time(s) per week | | | | | | | □ | E7 |
| Quantity per consumption | <50g; 50-100g; >100g | | | | | | | □ | E8 |
| 1. Do you eat egg? | No； _____ time(s) per week | | | | | | | □ | E9 |
| Quantity per consumption | <50g; 50-100g; >100g | | | | | | | □ | E10 |
| 1. Do you drink milk? | No； _____ time(s) per week | | | | | | | □ | E11 |
| Quantity per consumption | _____ml every time (a box = 200 ml, a cup = 160 ml) | | | | | | | □ | E12 |
| 1. Do you eat fruits? | No； _____ time(s) per week | | | | | | | □ | E13 |
| Quantity per consumption | 50-150g; 150-250 g; >250 g | | | | | | | □ | E14 |
| 1. Do you eat vegetable? | No； _____ time(s) per week | | | | | | | □ | E15 |
| Quantity per consumption | < 250g; 250 – 500 g; >500 g | | | | | | | □ | E16 |
| 1. Do you eat soy products? | No； _____ time(s) per week | | | | | | | □ | E17 |
| Quantity per consumption | 50 – 150 g; 150 – 250 g; >250 g | | | | | | | □ | E18 |
| 1. Daily intake of staple food? | 50 – 150 g; 150 – 250 g; >250 g | | | | | | | □ | E19 |
| 1. Do you drink coffee? | No； _____ time(s) per week | | | | | | | □ | E20 |
| 1. Do you drink tea? | No； Yes | | | | | | | □ | E21 |
| 1. Do you consume royal jelly? | No; Rarely; Often | | | | | | | □ | E22 |
|  | Years of consumption □□ | | | | | | |  | E23 |
| Lifestyle survey | | | | | | | |  |  |
| 1. Smoke | Never; Rarely; Often; Quitted | | | | | | | □ | F1 |
| If often smoke | Smoke for ____ years | | | | □□ | | | | F2 |
|  | ____ cigarettes per day | | | | □□ | | | | F3 |
| If quitted smoking | Quitted for ____ years | | | | □□ | | | | F4 |
| 1. Drinking white wine | No; <1 time/week; 1-3 times/week; >3 times/week | | | | | □ | | | F5 |
| 1. Drinking bear | No; <1 time/week; 1-3 times/week; >3 times/week | | | | | □ | | | F6 |
| 1. Drinking red wine | No; <1 time/week; 1-3 times/week; >3 times/week | | | | | □ | | | F7 |
| 1. How many times a week you eat out | ____ time(s)/week | | | | | □ | | | F8 |
| How many household members | ____ people | | | | | □ | | | F9 |
| Annual household cooking oil consumption | _____ L | | | □□ | | | | | F10 |
| 1. What salt do you eat at home | Regular salt; Low sodium salt; Regular salt mix with low sodium salt; Others (high zinc/selenium salt) | | | | | | | □ | F11 |
| 1. Average daily sleep | >8 h; 7-7.9 h; 6-6.9 h; 5-5.9 h; <5 h | | | | | | | □ | F12 |
| Is there a lack of deep sleep | No; Slight; Heavy | | | | | | | □ | F13 |
| Trouble falling asleep | No; Slight; Heavy | | | | | | | □ | F14 |
| 1. Eating habits | Light; Salty; Sweety; Others | | | | | | | □ | F15 |
